# Influence of ceramic waste powder on shear performance of environmentally friendly reinforced concrete beams

**DOI:** 10.1038/s41598-024-59825-7

**Published:** 2024-05-06

**Authors:** Yasin Onuralp Özkılıç, Essam Althaqafi, Alireza Bahrami, Ceyhun Aksoylu, Memduh Karalar, Nebi Özdöner, Evgenii M. Shcherban, Sergey A. Stel’makh, Alexey Beskopylny, Blessen Skariah Thomas

**Affiliations:** 1 Department of Civil Engineering, Faculty of Engineering, Necmettin Erbakan University, Konya, 42000 Turkey; 2 Department of Civil Engineering, Lebanese American University, Byblos, Lebanon; 3 Civil Engineering Department, College of Engineering, King Khalid University, 61421 Abha, Saudi Arabia; 4 Department of Building Engineering, Energy Systems and Sustainability Science, Faculty of Engineering and Sustainable Development, University of Gävle, 801 76 Gävle, Sweden; 5 Department of Civil Engineering, Faculty of Engineering and Natural Sciences, Konya Technical University, 42075 Konya, Turkey; 6 Department of Civil Engineering, Faculty of Engineering, Zonguldak Bulent Ecevit University, Zonguldak, 67100 Turkey; 7 Department of Transport Systems, Faculty of Roads and Transport Systems, Don State Technical University, 344003 Rostov-On-Don, Russia; 8 Department of Unique Buildings and Constructions Engineering, Don State Technical University, Gagarin Sq. 1, 344003 Rostov-On-Don, Russia; 9 Department of Engineering Geology, Bases, and Foundations, Don State Technical University, 344003 Rostov-On-Don, Russia; 10 Department of Civil Engineering, National Institute of Technology Calicut, Kerala, 673601 India

**Keywords:** Recycled, Ceramic waste powder, Reinforced concrete beam, Load-carrying capacity, Displacement, Stiffness, Civil engineering, Materials science, Structural materials, Mechanical properties

## Abstract

This investigation considered the usability of ceramic waste powder (CWP) in altered quantities in reinforced concrete beams (RCBs). In this way, it was aimed to reduce the environmental impacts of concrete by using CWP as a raw material in RCBs. 12 small-scale shear RCBs with the dimensions of 100 × 150 × 1000 mm were tested in this study. The variations of stirrups spacing and CWP ratio were examined in these specimens. The percentages of CWP by weight utilized in RCBs were 10%, 20%, and 30%, and stirrups spacings were adopted as 270 mm, 200 mm, and 160 mm. At the end of the study, it was determined that more than 10% CWP additive negatively affected the RCBs' compressive strength. The load-carrying capacity reduced between 30.3% and 59.4% when CWP increased from 0% to 30% as compared to RCB with stirrups spacing of 270 mm without CWP. However, compared to RCB with stirrups spacings of 200 mm and 160 mm without CWP, there were decreases in the load-carrying capacity as 21.4%–54.3% and 18.6%–54.6%, respectively. While the CWP ratio increased, the specimens with 160 mm, 200 mm, and 270 mm stirrups spacings obtained a lower maximum load value. However, with the increase of the CWP ratio in the specimens with 160 mm stirrups spacing, RCBs reached the maximum load-carrying capacity at an earlier displacement value. When stirrups spacing was selected as 270 mm, it was observed that the maximum load-carrying capacity of RCBs reached at a similar displacement value as the CWP ratio increased. Besides, it was resulted that the bending stiffness of RCBs reduced as the quantity of CWP enhanced. The bending stiffness decreased by 29.1% to 66.4% in the specimens with 270 mm stirrups spacing, 36.3% to 20.2% with 200 mm stirrups spacing, and 10.3% to 36.9% with 160 mm stirrups spacing. As an implication of the experiments, the use of CWP up to 10% in RCBs was realized as an economical and environmental approach and is suggested. There is some evidence to report that making use of CWP may be considered to be ecologically benign. This is due to the fact that reusing CWP may significantly reduce CO_2_ emissions, save energy, and reduce total power consumption. Furthermore, the experimental results were compared to the analytical calculations.

## Introduction

Recently, altered types of wastes have been utilized instead of raw construction materials^1–5^. Since one of the most used construction materials is concrete, this led researchers to improvement in the concrete technology^6–10^. Therefore, it is important to employ eco-friendly concrete with various approaches^11–14^. This can be summarized as tire waste^15–20^, sanitary ware waste^21–25^, glass waste^26–31^, fire clay^32^, marble dust^33–35^, ground granulated blast furnace slag^36,37^, waste fire clay^38–40^, granite waste^41–43^, red mud^44–47^, and polymer type waste^48–54^. The ceramic tiles business produces ceramic waste powder (CWP) as a byproduct during the final polishing process. The presence of CWP may result in the contamination of land, water, and air. The use of CWP as a substitute element in concrete will benefit the environment^55,56^. Furthermore, consumption of CWP in several manufacturing areas, mainly building, agricultural science, glass, and paper productions, would aid in caring for the environment. In addition, it is important to improve eco-friendly concrete by using CWP. Research works have indicated that ceramic production has an important place worldwide. In India, this rate is more than 100 million tons per year on a production basis. About 15%–30% of waste material in ceramic manufacturing is produced from the whole manufacture^57^. This waste is not reprocessed in any method at present. Nevertheless, CWP is strong, durable, and extremely unaffected by organic, chemical, and dreadful physical conditions^57^. As CWP stacks up daily, there is pressure on ceramic productions to the invention resulting from its throwing away. While CWP interacts with groundwater, it reasons serious health complications^58^. Several investigators are directing the use of this CWP in the improvement of concrete. Some investigations^59–62^ proffered that ceramic supplies have durable resistance in contrast to forces of biodegradation. As an implication of the extraordinary substances of crystalline aluminum and silica in ceramics, it is noticed that these are useful as supplementary cement for enhancing the strong point and stability performance of connectors and concrete prepared by ceramics^63,64^. With the objective of mitigating environmental issues, the use of CWP as a primary material in reinforced concrete beams (RCBs) is pursued. In the literature, some research used CWP to improve concrete. Kasi and Malasani^65^ examined the flexural performance of concrete. For this purpose, brick from the destruction waste was used as coarse aggregates in concrete after exposure to high temperatures. The reused brick aggregates were exchanged for granite aggregates by up to 25% in its volume to create reused brick aggregate concrete. Moreover, the beam samples of 100 × 100 × 500 mm were elected to evaluate the flexural strength. It was found that concrete with recycled aggregates exhibited better performance than concrete with granite aggregates in terms of the flexural strength at elevated temperatures. Debieb and Kenai^66^ exchanged coarse aggregates with crushed brick aggregates at 25%, 50%, 75%, and 100%. It was reported that using 25% crushed bricks as replacement of coarse aggragates was logical based on the flexural strength capacity. A decrease in the flexural strength of about 15% was detected in crushed brick aggregate concrete. Another evaluation was made on the shear strength of RCBs prepared with recycled brick aggregates by Mohammed et al.^67^. For this purpose, 32 RCBs of the sizes 200 × 300 × 2100 mm and 200 × 300 × 2400 mm were prepared to assess the shear strength of RCBs estimated via altered codes and rupture mechanics approaches. They concluded that RCBs prepared with recycled brick aggregates demonstrated similar shear strength as RCBs prepared with virgin brick aggregates. Furthermore, current code provisions can be utilized to expect the shear strength of RCBs prepared with recycled brick aggregates. Said et al.^68^ examined the shear performance of reinforced mortar beams using polyvinyl alcohol fiber in varying amounts from 0% to 2.25%, together with fly ash (55%) and silica fume (15%). A finite element model was created to predict the fracture pattern, load–deflection, energy absorption, and shear strength of 14 beams experimentally evaluated under two intense loads. It was found that the fracture pattern and ductility of the tested beams improved by utilizing polyvinyl alcohol fiber. Shaaban et al.^69^ employed several types of fibers in RCBs specifically intended to fail under shear. Additionally, a practical equation was used to predict the magnitude of the shear strength. It was witnessed that the anticipated ultimate shear strength, obtained by the derived equation, exhibited a remarkable concurrence with the experimental data, displaying a clear linear connection characterized by a strong correlation. The effects of the curing and drying regime on the mechanical qualities and permeation properties of concrete containing steel fibers and crumbed rubber extracted from scrap tires were investigated by Shaaban et al.^70^. It was resulted that cracking was seen before failure at the maximum load. Aksoylu et al.^56^ examined the bending behavior of RCBs with CWP. RCBs were desgined to fail in bending. The results showed that using CWP in RCBs is an eco-friendly solution.

As proffered above, numerous examinations of concrete performance with CWP are in the literature without any reinforcement details. Moreover, there are minimal investigations on the impact of the CWP percentage, replacing cement. Nonetheless, in the literature, no studies achieved the effects of altered rates of stirrups reinforcements and CWP on the RCBs' rupture and shear behavior. The main objective of this experimental study was to determine how different percentages of CWP affect the RCBs' shear performance. This investigation evaluated the possibility of the usage of CWP in real practice, as in beam members. As an implication, this study significantly supports the literature and engineering practice.

## Materials and methods

**Figure 1:**
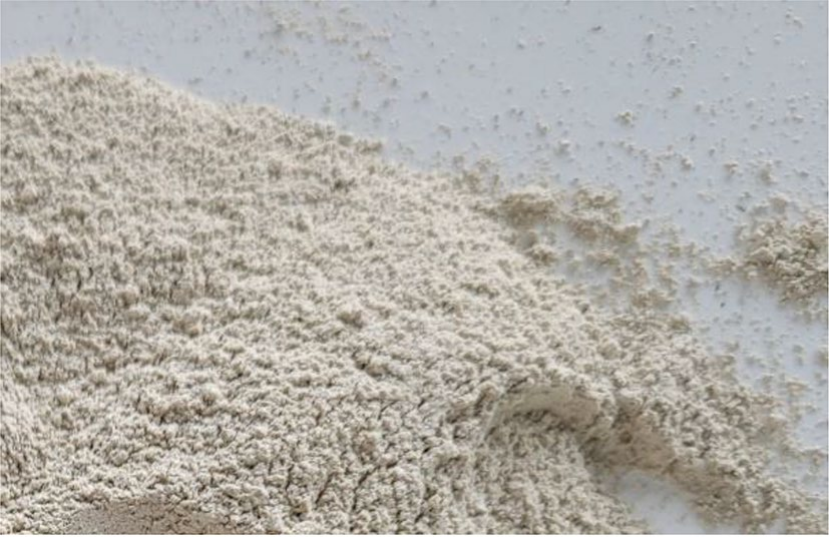
CWP.

**Figure 2:**
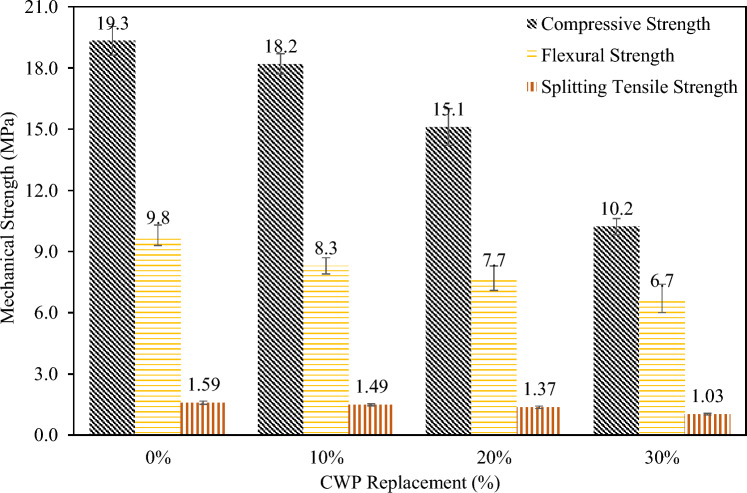
Mechanical strengths of samples with and without CWP.

In this study, to make eco-friendly RCBs, CWP replaced some portions of cement. A fixed mixture design (Table 1) with changing the cement ratio was used. CWP was employed as 10%, 20%, and 30% of the cement weight. The utilized CWP is presented in Fig. 1. The mechanical strengths of the samples are illustrated in Fig. 2. A slight decrease in the mechanical strengths was observed when 10% of CWP was utilized to replace cement. However, this reduction significantly grew after 10% of CWP.

**Table 1:** Mixture design of RCBs.

CWP	Cement (kg/m^3^)	Water (kg/m^3^)	Water/Cement	Fine aggregate (kg/m^3^)	Coarse aggregate (kg/m^3^)	CWP (kg/m^3^)
0%	580	270	0.48	785	900	0
10%	522		0.52			58
20%	464		0.58			116
30%	406		0.66			174

**Figure 3:**
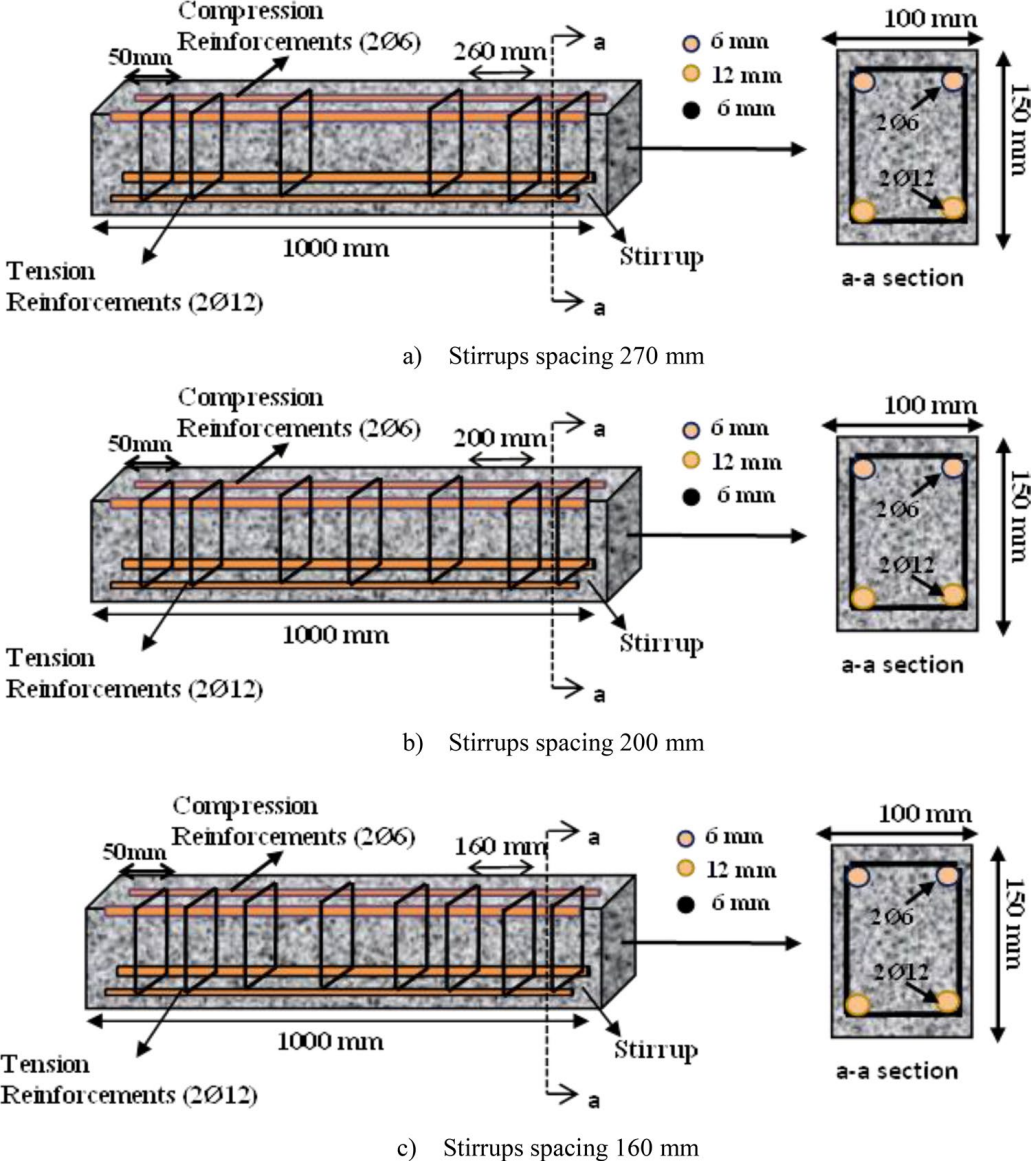
Reinforcement layout for specimens.

**Figure 4:**
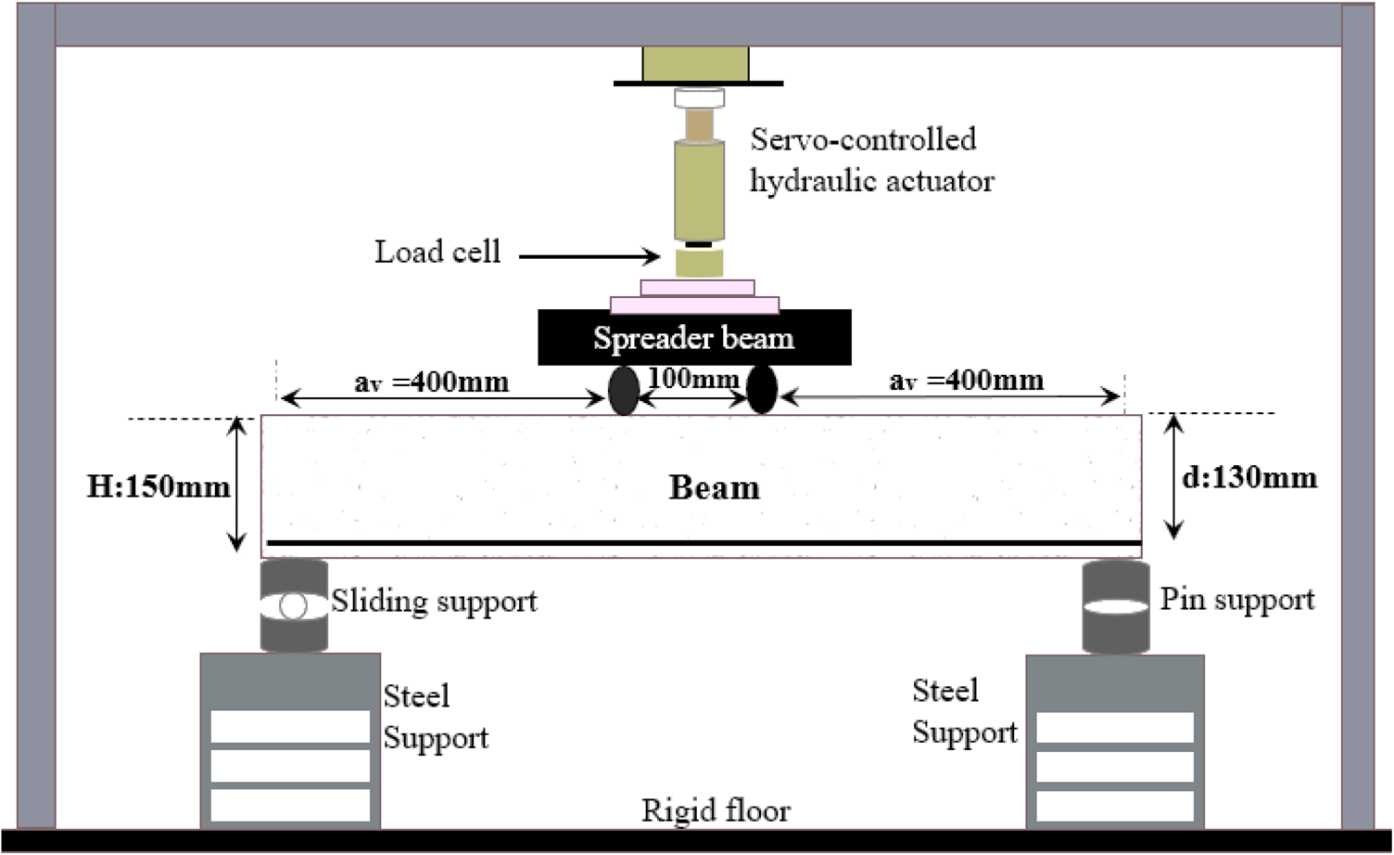
Test setup of specimens.

A shear collapse without any prior warning is likely to happen abruptly in a beam that lacks appropriately designed shear reinforcements when it is overloaded to the point of failure (brittle failure). Therefore, to ensure that flexural damage would occur before the shear failure, concrete must be given with special shear reinforcements. Through the current experimental program, the impacts of stirrups spacing on the shear behavior and shear capacity of RCBs with minimal shear reinforcements were examined. In other words, the influence of stirrups on the shear-carrying capacity in the tested RCBs was significant and might hinder the observation of the effect of CWP. Consequently, the contribution of CWP was tried to be understood by considering different stirrups spacings. Additionally, as the CWP percentages changed, RCBs were tested to assess the effectiveness of the changes in their performance. In this way, the ductility, stiffness, and energy dissipation of the specimens were evaluated with respect to the stirrups spacings and CWP percentages. To achieve this objective, the load-carrying capacities of RCBs were assessed. The spacings between stirrups were set at 160 mm, 200 mm, and 270 mm, while the specimens were tested with varying percentages (0%, 10%, 20%, and 30%) of the CWP additions. The results were then compared to those of the reference specimens. The experimental test specimens are depicted in Fig. 3. A total of 12 RCBs were produced to examine the shear capacity of the CWP-contributed beams. This study investigated the RCBs' shear performance using CWP concrete mixture in certain proportions instead of cement. The size of the specimens was planned as 100 × 150 × 1000 mm. In addition, as can be seen in Fig. 4, each specimen was evaluated under four-point bending tests. Altered main parameters were considered to assess the implications of CWPs on the shear performance, weight ratios of CWP, and spacings of stirrups. The weight percentages of 0%, 10%, 20%, and 30% CWP were used, while stirrups spacings of 270 mm, 200 mm, and 160 mm were considered. The longitudinal reinforcements utilized for the tension and compression sections were selected as 2Ø12 and 2Ø6, respectively, as indicated in Fig. 3. The specifications of the test samples are listed in Table 2.

**Table 2:** Features of specimens.

No.	Test samples	Stirrups diameter/Spacing	Volumetric ratio of stirrups (%)	Percentage of CWP
1	S-REF#1	6/270	2.1	0
2	S-REF#2	6/200	2.8	0
3	S-REF#3	6/160	3.53	0
4	S-CERAMIC#1	6/270	2.1	10
5	S-CERAMIC#2	6/200	2.8	10
6	S-CERAMIC#3	6/160	3.53	10
7	S-CERAMIC#4	6/270	2.1	20
8	S-CERAMIC#5	6/200	2.8	20
9	S-CERAMIC#6	6/160	3.53	20
10	S-CERAMIC#7	6/270	2.1	30
11	S-CERAMIC#8	6/200	2.8	30
12	S-CERAMIC#9	6/160	3.53	30

## Experimental investigation of RCBs

### Efficiency of altered stirrups spacings

In this section, RCBs were tested with altered stirrups spacings to examine the efficiency of the stirrups. Details are presented in the following.

#### Case 1: Rupture and load–displacement form of RCBs (S-REF#1, S-REF#2, and S-REF#3)

**Figure 5:**
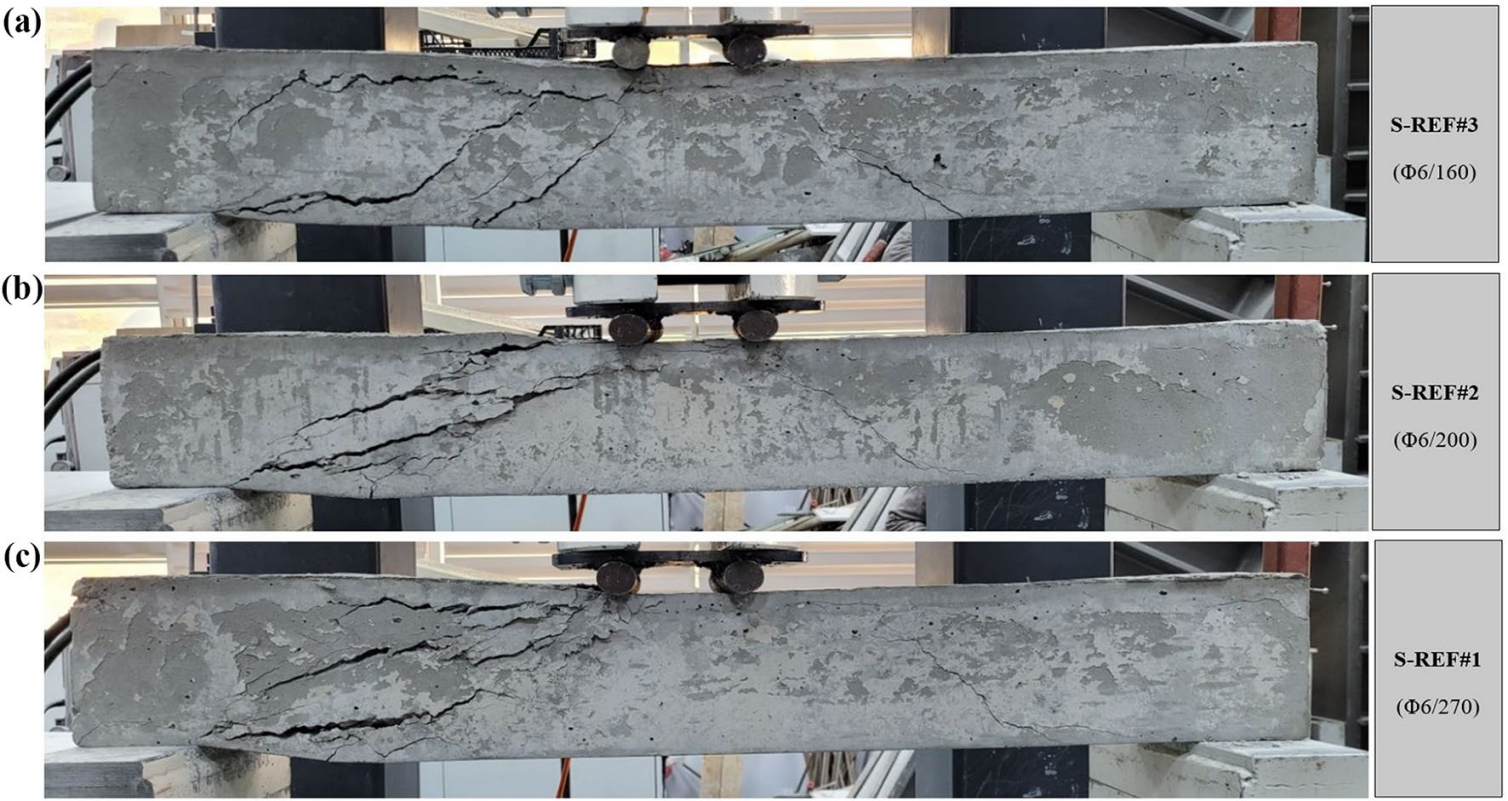
Failure patterns of RCBs with 0% CWP and stirrups spacings of: **a**) 160 mm, **b**) 200 mm, and **c**) 270 mm.

**Figure 6:**
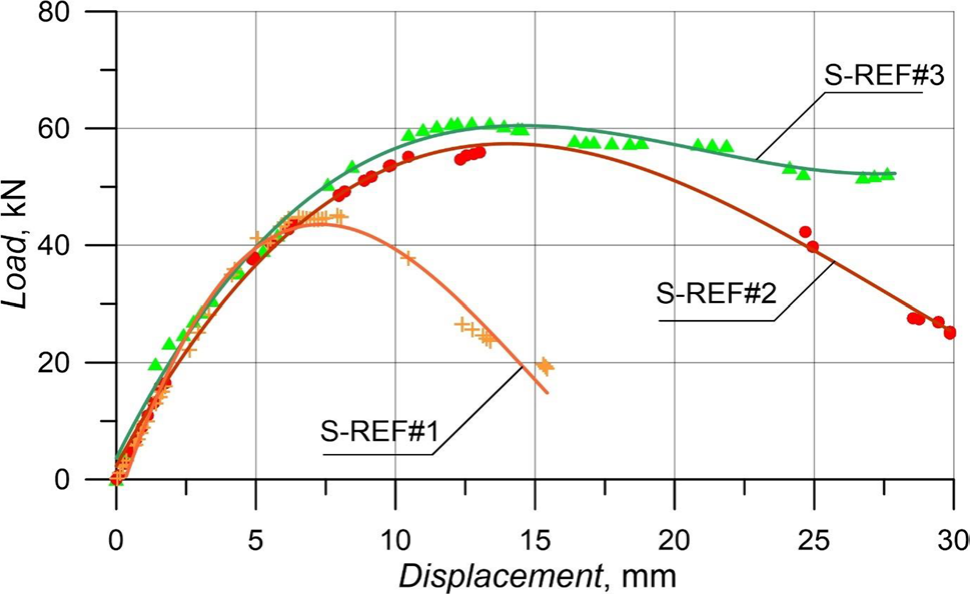
Load–displacement results of RCBs with different spacings of stirrups.

As proffered in Figs. 5 and 6, the failure patterns and load–displacement diagrams of the RCB tests are observed, respectively. In Fig. 4, the maximum load-carrying capacity of S-REF#3 with 160 mm stirrups spacing was obtained as 61.03 kN, while the displacement at *P*_max_ was 13.10 mm. The maximum load-carrying capacity value and displacement at *P*_max_ of S-REF#2 and S-REF#1 specimens, in which the stirrups spacings were increased to 200 mm and 270 mm, were 55.88 kN and 13.01 mm and 45.11 kN and 7.92 mm, respectively. As a result, the specimens suffered from the shear damage earlier in the load-carrying capacity due to the increased stirrups spacings. Compared to S-REF#3, this ratio was 8.4% lower in S-REF#2 and 26% lower in S-REF#1. According to Fig. 5, all three specimens experienced the shear damage and collapsed.

#### Case 2: Rupture and load–displacement form of RCBs (S-CERAMIC#1, S-CERAMIC#2, and S-CERAMIC#3)

**Figure 7:**
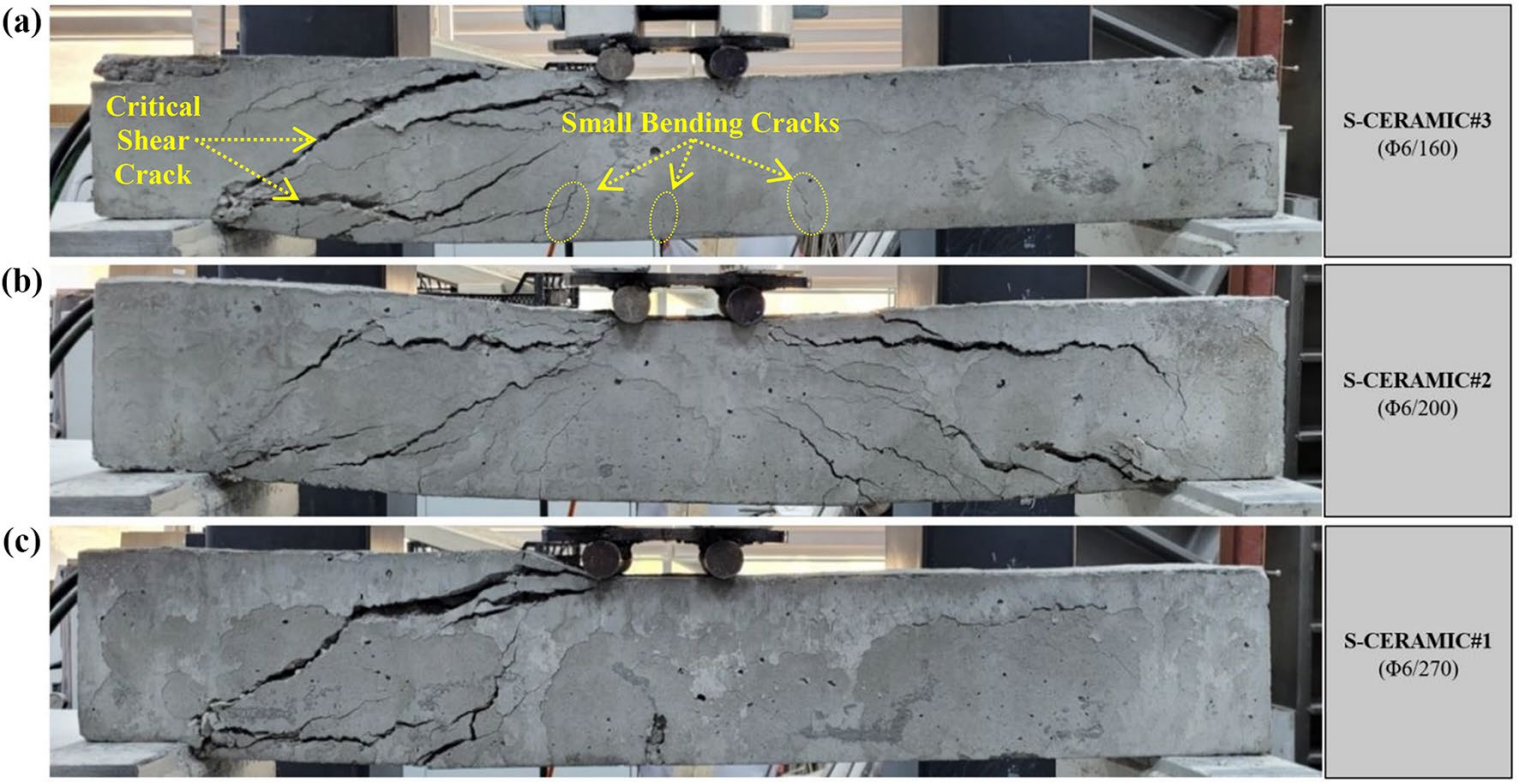
Failure patterns of RCBs with 10% CWP and stirrups spacings of: **a**) 160 mm, **b**) 200 mm, and **c**) 270 mm.

**Figure 8:**
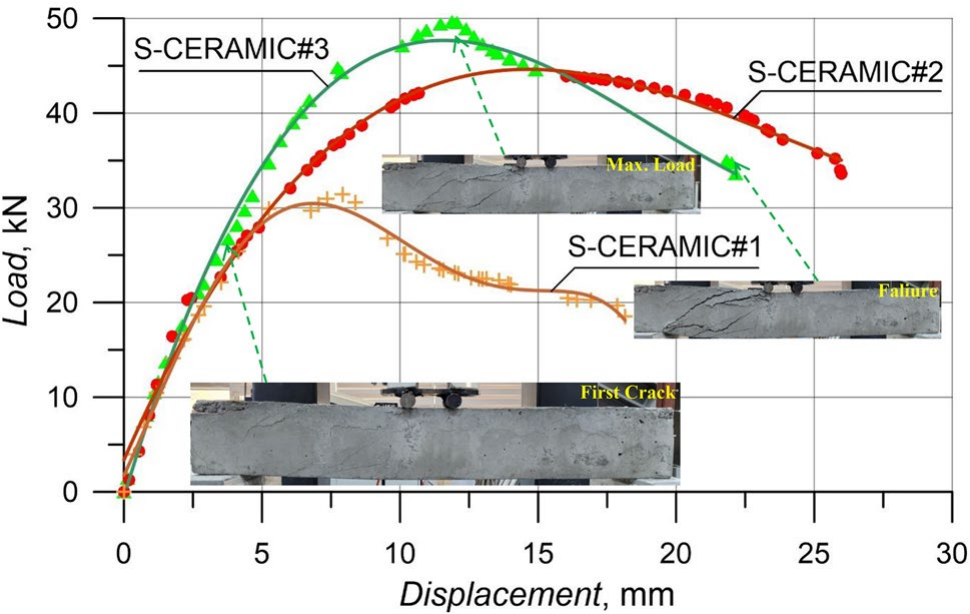
Load–displacement results of RCBs with different spacings of stirrups.

In this section, 10% CWP was used as a replacement for cement to evaluate the effects of CWPs with spacings of stirrups on the shear performance of RCBs. Figures 7 and 8 demonstrate the RCBs' end-of-experiment failure views and load–displacement curves with the 10% CWP content. According to Fig. 8, the highest load and *P*_max_ displacement recorded by S-CERAMIC#3 were 49.65 kN and 11.88 mm, respectively. In S-CERAMIC#2 and S-CERAMIC#1 samples, these values were found to be 43.83 kN and 16.03 mm and 31.43 kN and 7.78 mm, respectively, in comparison to S-CERAMIC#3. Hence, S-CERAMIC#2 and S-CERAMIC#1 samples carried 11.6% and 36.6% less loads than S-CERAMIC#3, respectively. Compared to Case 1, 10% CWP contribution led to a reduction in the load-carrying capacity in RCBs with all three stirrups spacings. Similar to Case 1, all 10% of CWP-contributed specimens suffered from the shear damage at the end of the tests. The curves provide a detailed representation of the crack formations in RCBs (Fig. 8).

#### Case 3: Rupture and load–displacement form of RCBs (S-CERAMIC#4, S-CERAMIC#5, and S-CERAMIC#6)

**Figure 9:**
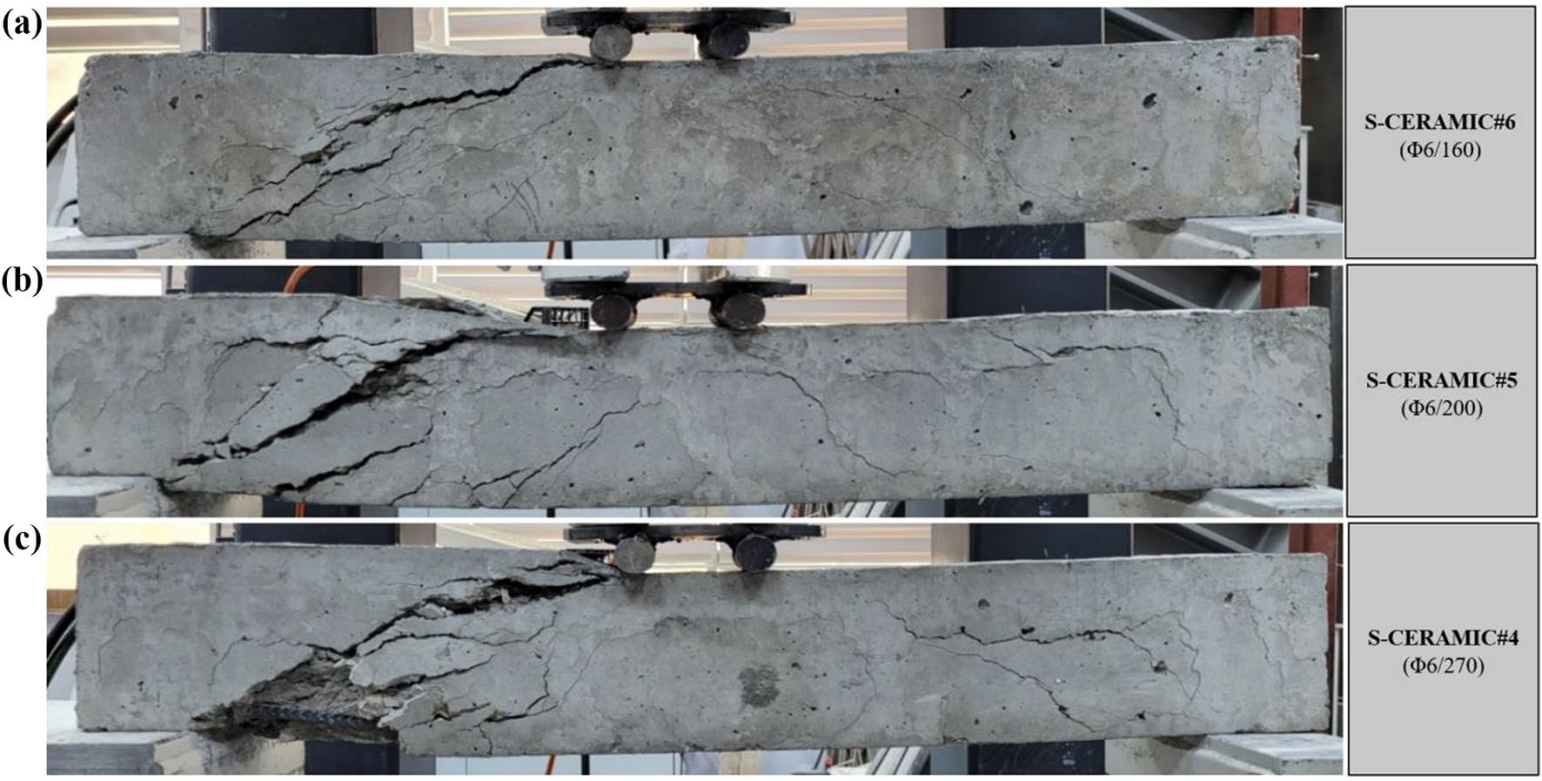
Failure patterns of RCBs with 20% CWP and stirrups spacings of: **a**) 160 mm, **b**) 200 mm, and **c**) 270 mm.

**Figure 10:**
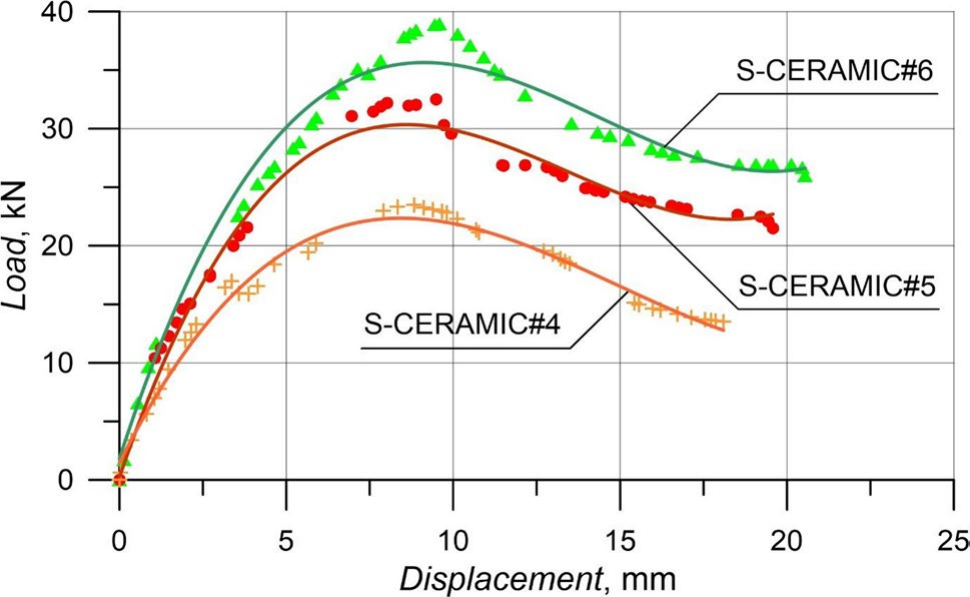
Load–displacement results of RCBs with different spacings of stirrups.

The failure patterns and load–displacement relations of the samples with 20% CWP ratio instead of the cement amount by weight are displayed in Figs. 9 and 10. In Fig. 9, the specimens had the shear damage at the end of the experiment, similar to Case 1 and Case 2, and collapsed. The rise in the CWP ratio resulted in a decline in the load-carrying capacity. Based on Fig. 10, S-CERAMIC#6 specimen with 160 mm stirrups spacing had 38.94 kN maximum load and 9.58 mm displacement corresponding to *P*_max_. Besides, 32.47 kN and 9.49 mm and 23.51 kN and 8.69 mm were found for S-CERAMIC#5 and S-CERAMIC#4 specimens, respectively, with 200 mm and 270 mm stirrups spacings. S-CERAMIC#5 and S-CERAMIC#4 specimens carried 16.6% and 39.6% less loads than S-CERAMIC#6. These consequences were noticed to be similar to Case 1 and Case 2.

#### Case 4: Rupture and load–displacement form of RCBs (S-CERAMIC#7, S-CERAMIC#8, and S-CERAMIC#9)

**Figure 11:**
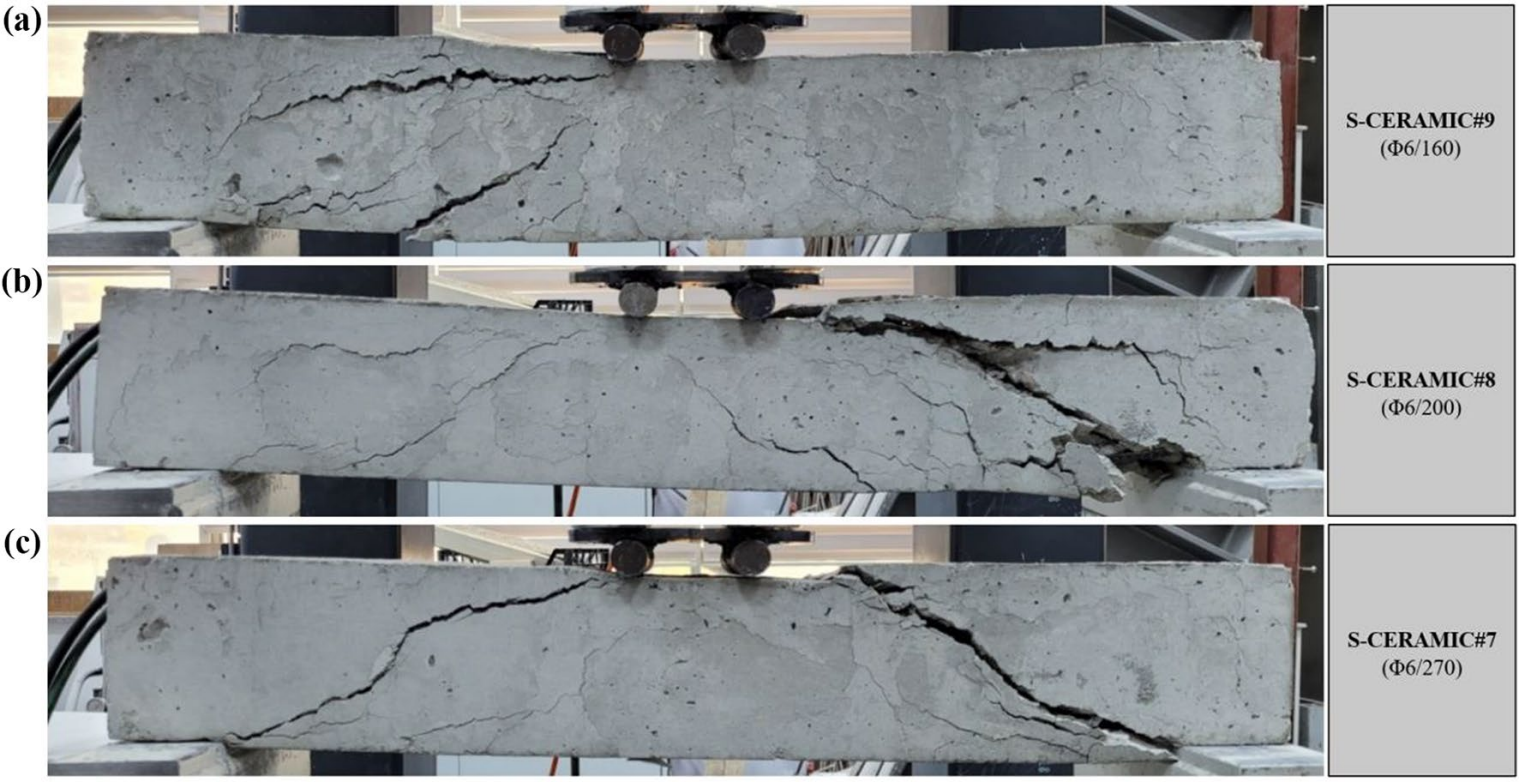
Failure patterns of RCBs with 30% CWP and stirrups spacings of: (**a**) 160 mm, (**b**) 200 mm, and (**c**) 270 mm.

**Figure 12:**
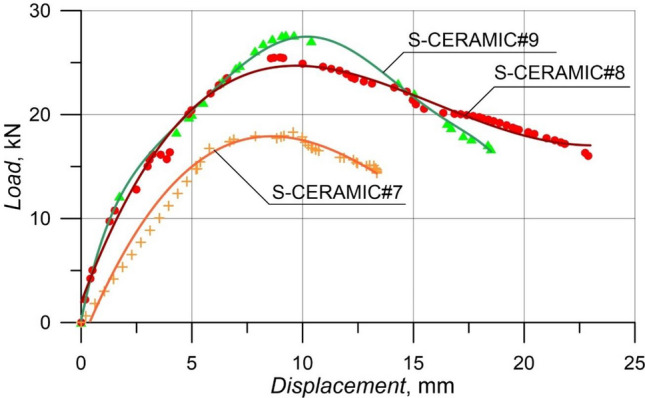
Load–displacement results of RCBs with different spacings of stirrups.

This section investigates the variation of the shear capacity by adding 30% CWP to RCBs with different stirrups spacings. The end-of-experiment damage and load–displacement relationship of each specimen are presented in Figs. 11 and 12, respectively. In Fig. 12, S-CERAMIC#9 specimen suffered from the sudden shear damage after reaching 27.68 kN. The *P*_max_ displacement was obtained as 9.42 mm. Two specimens, S-CERAMIC#8 and S-CERAMIC#7, were tested with stirrups spacings of 200 mm and 270 mm, respectively. S-CERAMIC#8 specimen had a load capacity of 25.51 kN and a *P*_max_ displacement of 8.84 mm, while S-CERAMIC#7 specimen had a load capacity of 18.29 kN and a *P*_max_ displacement of 9.57 mm. Consequently, the rise in the CWP rate to 30% resulted in a decline in the load-carrying capacity. Moreover, it is shown that the load–displacement capacities of RCBs expanded gradually as stirrups spacing decreased. This suggests that stirrups spacing had an efficient effect on the load–displacement capacities of RCBs. It was noticed that these results were similar to those in Case 1, Case 2, and Case 3. However, the increase in the CWP ratio caused the maximum load values of RCBs to approach each other. When Figs. 6, 8, 10, and 12 are evaluated, it is clear that the reference specimens had a maximum load-carrying capacity for all stirrups spacings. As the CWP ratio decreased, the load values were obtained closer to the reference specimens. In other words, in the RCB specimens with spacings of stirrups as 160 mm, 200 mm, and 270 mm, the CWP additives as 0%, 10%, 20%, and 30% reduced the load-carrying capacities of RCBs compared to the reference specimens, respectively, as 18.6%, 36.1%, and 54.6% (for 160 mm stirrups spacing), 21.4%, 41.8%, and 54.3% (for 200 mm stirrups spacing), and 30.3%, 47.8, and 59.4% (for 270 mm stirrups spacing). Furthermore, although this reduction in the load-carrying capacity of RCBs was related to the quantity of CWP, it was not linear. In addition, the load-carrying capacity decreased as stirrups spacing increased. As stirrups spacing in the samples decreased (270 mm > 200 mm > 160 mm), the load-carrying capacity was negatively affected by rising rates of CWP (10% < 20% < 30%).

### Efficiency of altered percentages of CWP

RCBs were also tested with altered CWP percentages to assess their efficiency on the performance of RCBs. For this purpose, the weight percentages of 0%, 10%, 20%, and 30% CWP were adopted and tested.

#### Case 1: Rupture and load–displacement form of RCBs with different percentages of CWP and spacing of stirrups as 160 mm

**Figure 13:**
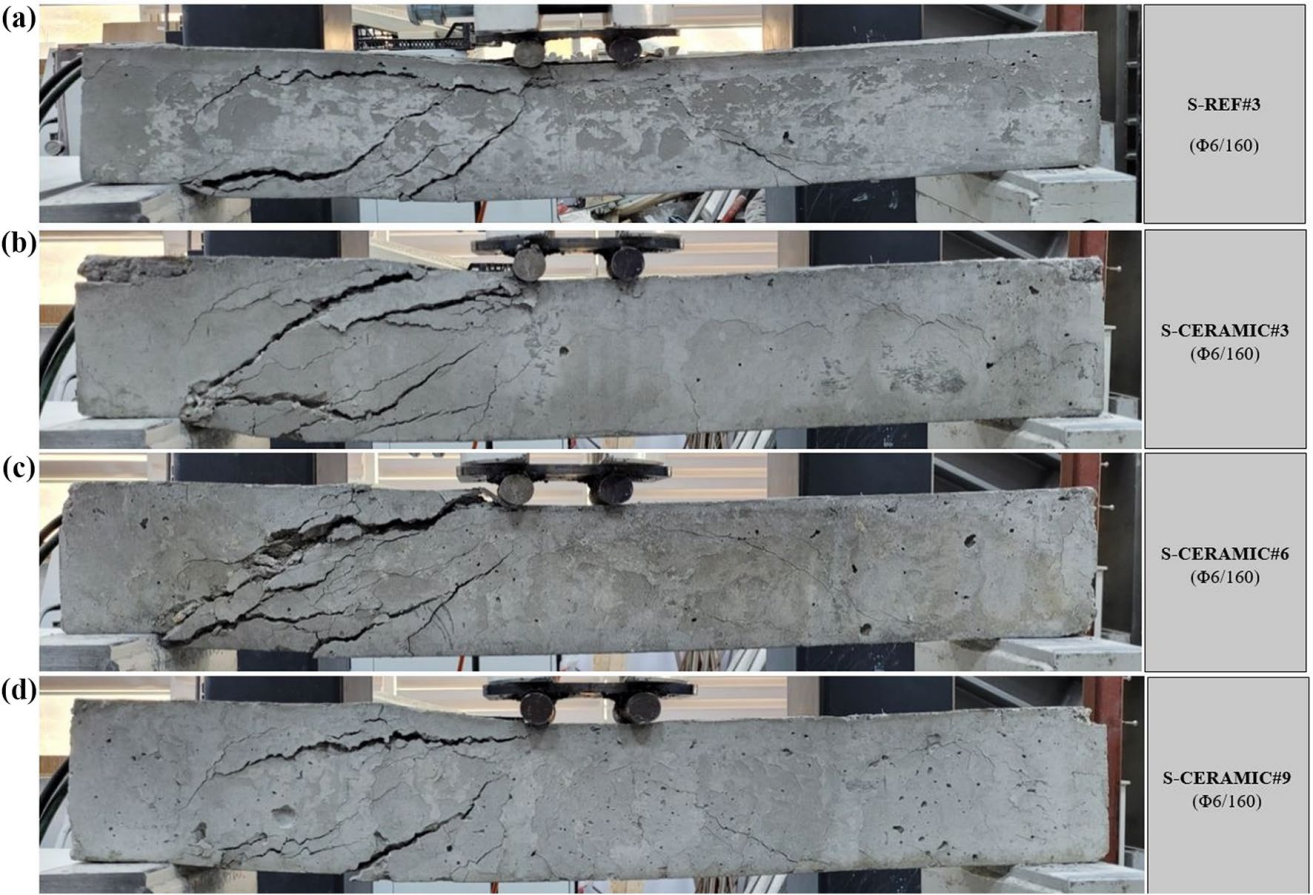
Failure patterns of RCBs with stirrups spacing of 160 mm and CWP percentages of: (**a**) 0%, (**b**) 10%, (**c**) 20%, and (**d**) 30%.

**Figure 14:**
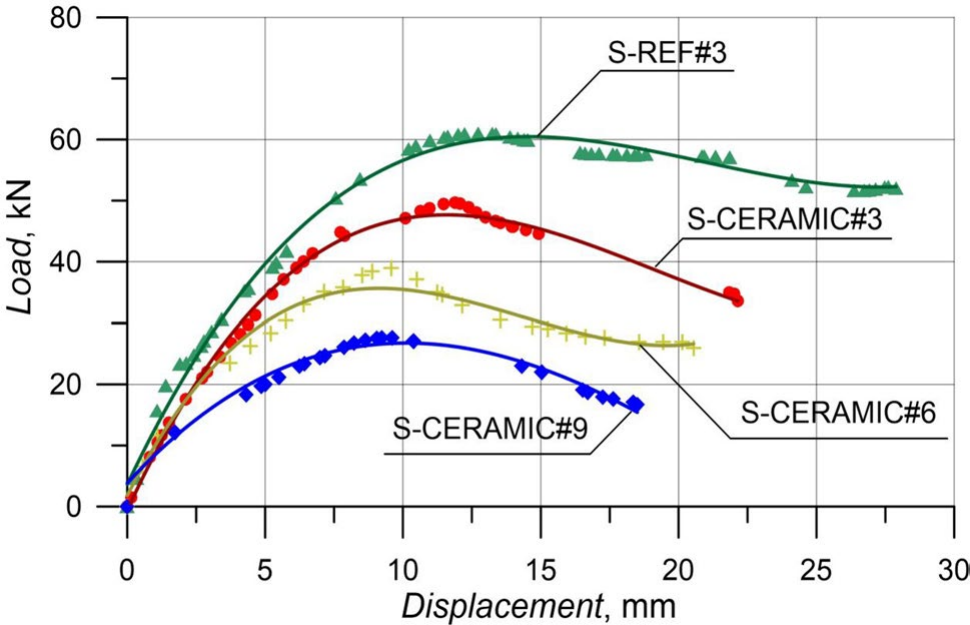
Load–displacement results of RCBs with stirrups spacing of 160 mm and altered quantity of CWP.

The evaluation of RCBs with constant stirrups spacing and different CWP ratios is explained in this section. The amounts of CWP were taken as 0%, 10%, 20%, and 30% while stirrups were constantly spaced 160 mm apart. The failure modes of  RCBs are depicted in Fig. 13. Based on Fig. 14, the maximum load was acquired as 61.03 kN, and the *P*_max_ displacement was achieved as 13.10 mm when the CWP ratio was set to 0% (S-REF#3). For a CWP ratio of 10% (S-CERAMIC#3), the maximum load and *P*_max_ displacement were 49.65 kN and 11.88 mm, respectively. The maximum load and *P*_max_ displacement were found to be 38.94 kN and 9.58 mm, respectively, when the CWP ratio increased to 20% (S-CERAMIC#6). After increasing the CWP ratio to 30% (S-CERAMIC#9), it was resulted that the maximum load and *P*_max_ displacement were reduced to 27.68 kN and 9.42 mm, respectively. However, the load-carrying capacity of S-CERAMIC#3, S-CERAMIC#6, and S-CERAMIC#9 specimens dropped by 18.6%, 36.1%, and 54.6%, respectively, in comparison to S-REF#3. This pointed out that increasing the CWP additive decreased the load-carrying capacity. In addition, depending on the increased CWP ratio, RCBs reached the maximum load value at an earlier displacement value. The findings revealed that the structural features of RCBs declined as the replacement rate of CWP reached 10% or higher. This was ascribed to a diluting effect and increased CWP porosity that negatively impacted the microstructure and properties of concrete (Rachied et al. 2023). The decrease in the strength is consistent with the dilution effect and increased porosity, which disrupt the concrete microstructure and hinder the strength growth.

#### Case 2: Rupture and load–displacement form of RCBs with different percentages of CWP and spacing of stirrups as 200 mm

**Figure 15:**
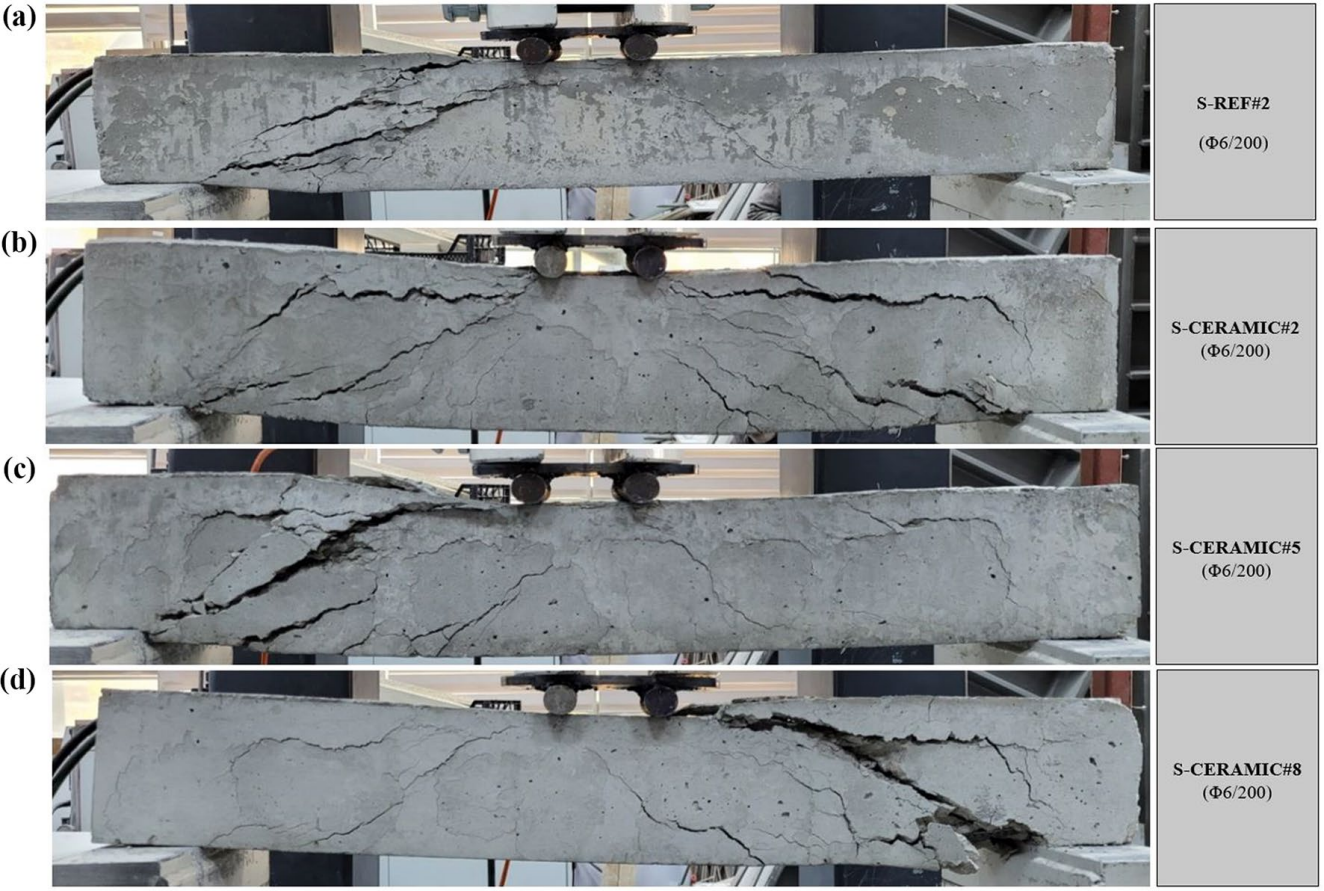
Failure patterns of RCBs with stirrups spacing of 200 mm and CWP percentages of: (**a**) 0%, (**b**) 10%, (**c**) 20%, and (**d**) 30%.

**Figure 16:**
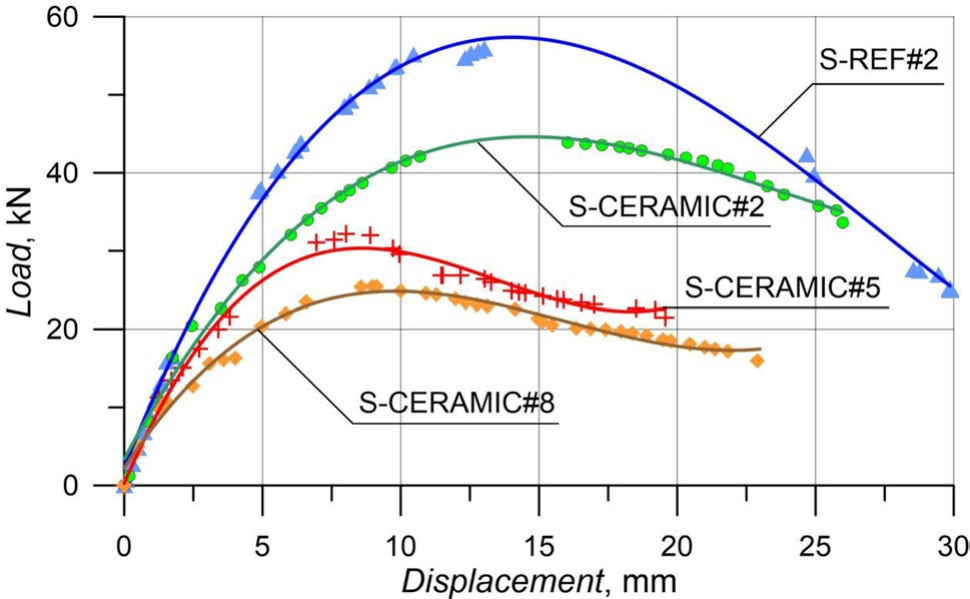
Load–displacement results of RCBs with stirrups spacing of 200 mm and altered quantity of CWP.

Here, spacing of stirrups in RCBs was constantly conisdered as 200 mm, while the amounts of CWP were 0%, 10%, 20%, and 30% to examine the efficiency of the altered CWP percentages. The failure patterns of the tested  RCBs with 0%, 10%, 20%, and 30% CWP are indicated in Fig. 15. As can be observed from Fig. 16, the *P*_max_ displacement measured was 13.01 mm, maximum load was 55.88 kN, and CWP ratio was 0% (S-REF#2). Where the 10% (S-CERAMIC#2) CWP ratio was chosen, the maximum load and *P*_max_ displacement were 16.03 mm and 43.87 kN, respectively. It was discovered that the maximum load and *P*_max_ displacement decreased to 32.47 kN and 9.49 mm, respectively when the CWP ratio rose to 20% (S-CERAMIC#5). This was followed by a decrease in the maximum load and *P*_max_ displacement to 25.51 kN and 8.84 mm, respectively, when the CWP ratio increased to 30% (S-CERAMIC#8). On the other hand, the load-carrying capacity of S-CERAMIC#2, S-CERAMIC#5, and S-CERAMIC#8 samples decreased by 21.4%, 41.8%, and 54.3%, respectively, when compared to S-REF#2. These findings demonstrated that the use of the CWP additive led to a greater reduction in the load-carrying capacity as the stirrups spacing increased. In addition, it was determined in Figs. 15 and 16 that the stiffness of the samples decreased and cracks increased with the increase of the CWP ratio.

#### Case 3: Rupture and load–displacement form of RCBs with different percentages of CWP and spacing of stirrups as 270 mm

**Figure 17:**
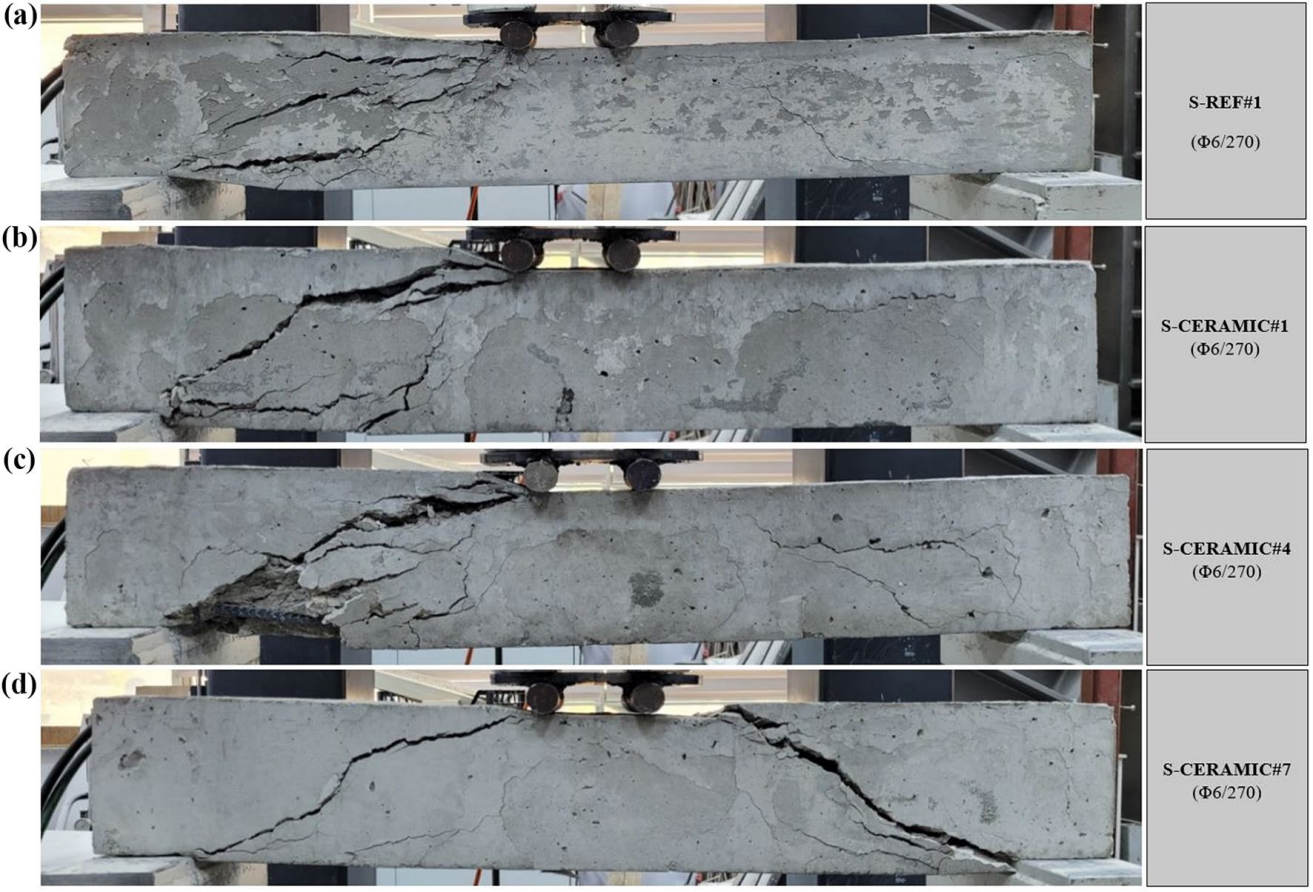
Failure patterns of RCBs with stirrups spacing of 270 mm and CWP percentages of: (**a**) 0%, (**b**) 10%, (**c**) 20%, and d) 30%.

**Figure 18:**
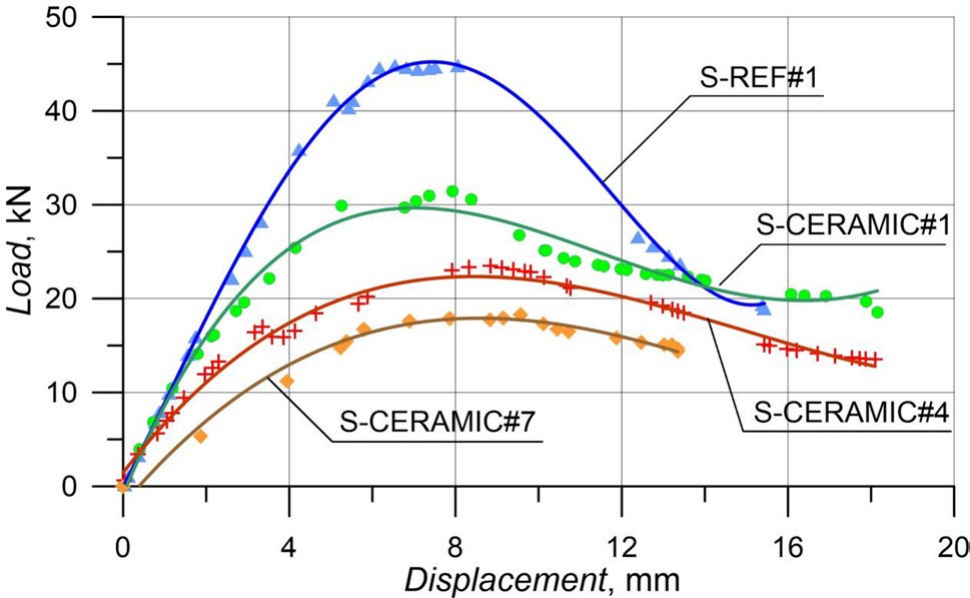
Load–displacement results of RCBs with stirrups spacing of 270 mm and altered quantity of CWP.

In order to evaluate the efficiency of an altered CWP percentage, the amounts of CWP were taken as 0%, 10%, 20%, and 30% when spacing of stirrups in RCBs was constant as 270 mm. The results of the experimental tests on RCBs with 0%, 10%, 20%, and 30% CWP are presented in Fig. 17. Based on Fig. 18, for the CWP ratio as 0% (S-REF#1), the maximum load was obtained as 45.11 kN and *P*_max_ displacement was found as 7.92 mm. The 10% (S-CERAMIC#1) CWP ratio resulted in the maximum load and *P*_max_ displacement of 31.43 kN and 7.78 mm, respectively. It was seen that the highest load and highest *P*_max_ displacement decreased to 23.51 kN and 8.69 mm when the CWP ratio rose to 20% (S-CERAMIC#4). Subsequently, when the CWP ratio increased to 30% (S-CERAMIC#7), it was observed that the highest load and *P*_max_ displacement decreased to 18.29 kN and 9.57 mm, respectively. To clarify, when comparing S-REF#1 to S-CERAMIC#1, S-CERAMIC#4, and S-CERAMIC#7 samples, the load-carrying capacity declined by 30.3%, 47.8%, and 59.4%, respectively. This point displays that when stirrups spacing increased more, the CWP additive dramatically lowered the load-carrying capacity. It was also shown that the samples reached their maximum load-carrying capacity at approximately similar displacement values with increasing stirrups spacing. When the CWP ratio increased, at the same time, a significant decrease was witnessed in the initial stiffness values. This illustrated that CWP was less effective on stiffness as the stirrups spacing increased.

As can be noticed from Figs. 14, 16, and 18, the load-carrying capacity of RCBs diminished as the CWP quantity increased from 0% to 30%. The maximum load value reached by the samples decreased as the CWP ratio increased in the samples with stirrups spacings of 160 mm, 200 mm, and 270 mm. However, with the increase of the CWP ratio in the samples with 160 mm stirrups spacing, the samples reached the maximum load-carrying capacity at an earlier displacement value. While the space between the stirrups was chosen as 270 mm, it was observed that the maximum load-carrying capacity of the samples reached at a similar displacement value as the CWP ratio increased. In addition, it was detected that CWP reduced the quantity and range of the flexural ruptures in RCBs.

### Efficiency of altered percentages of CWP on ductility, stiffness, and energy dissipation

The stiffness, ductility, and energy dissipation values of the specimens are assessed by considering Tables 3 and 4. Sufficient ductility could not be achieved in all test samples. In the literature, the ductility ratio of RCBs with under-reinforced design was given between 4 and 5. The ductility ratios obtained in Table 3 are below this limit. In addition, when the end-of-test damages of the samples are examined, it is understood that RCBs were generally subjected to the diagonal tension failure. The stiffness of the samples corresponding to the maximum load value decreased with increasing the CWP ratio.

**Table 3:** Experimental test results for stiffness and ductility values.

Test samples	*P*_max_ (kN)	Displacement at *P*_max_ (mm)	Stiffness at *P*_max_ (kN/mm)	*P*_u_ (0.85*P*_max_) (kN)	Displacement at 0.85*P*_max_, *δ*_y_ (mm)	Stiffness at 0.85*P*_max_ (kN/mm)	*δ*_u_ (mm)	Ductility ratio
S-REF#1	45.11	7.92	5.69	38.34	4.62	8.29	10.26	2.22
S-REF#2	55.88	13.01	4.29	47.49	7.65	6.20	20.21	2.64
S-REF#3	61.03	13.10	4.65	51.87	7.98	6.49	25.79	3.23
S-CERAMIC#1	31.43	7.78	4.03	26.71	4.39	6.07	9.54	2.17
S-CERAMIC#2	43.87	16.03	2.73	37.29	7.95	4.68	23.82	2.99
S-CERAMIC#3	49.65	11.88	4.17	42.20	6.98	6.04	17.04	2.44
S-CERAMIC#4	23.51	8.69	2.70	19.98	5.83	3.42	12.2	2.09
S-CERAMIC#5	32.47	9.49	3.42	27.60	5.81	4.75	11.05	1.90
S-CERAMIC#6	38.94	9.58	4.06	33.10	6.42	5.15	12.04	1.87
S-CERAMIC#7	18.29	9.57	1.91	15.54	5.41	2.87	12.33	2.27
S-CERAMIC#8	25.51	8.84	2.88	21.68	5.66	3.82	14.89	2.62
S-CERAMIC#9	27.68	9.42	2.93	23.52	6.50	3.61	13.77	2.11

**Table 4:** Experimental test results for energy dissipation capacities.

Test samples	Maximum displacement (mm)	Energy dissipation at *P*_max_ (kj)	Energy dissipation at 0.85*P*_max_ (kj)	Plastic energy dissipation (kj)	Total energy dissipation (kj)
S-REF#1	15.43	0.240	0.116	0.354	0.471
S-REF#2	29.86	1.076	0.234	1.010	1.244
S-REF#3	27.89	0.560	0.325	1.059	1.383
S-CERAMIC#1	18.15	0.172	0.087	0.323	0.410
S-CERAMIC#2	25.98	0.533	0.189	0.739	0.928
S-CERAMIC#3	22.15	0.406	0.204	0.624	0.828
S-CERAMIC#4	18.09	0.143	0.096	0.216	0.312
S-CERAMIC#5	19.57	0.221	0.146	0.322	0.468
S-CERAMIC#6	20.54	0.265	0.135	0.442	0.577
S-CERAMIC#7	13.34	0.122	0.049	0.127	0.176
S-CERAMIC#8	22.98	0.156	0.087	0.363	0.450
S-CERAMIC#9	18.50	0.177	0.108	0.272	0.379

The shear damages of the samples signify that they did not have sufficient energy dissipation capacity. However, the energy dissipation capacities for different levels were calculated and are presented in Table 4. The assessment can be made for the energy dissipation capacity corresponding to the maximum load level. As can be seen in Table 4, the energy dissipation capacity corresponding to the maximum load value decreased depending on the increase in stirrups spacing and CWP ratio. In the samples with the CWP ratios of 10%, 20%, and 30% and stirrups spacing of 270 mm, the respective reductions were determined as 28.3% (S-CERAMIC#1), 40.4% (S-CERAMIC#4), 49.1% (S-CERAMIC#7). The ratios exhibited an upward trend as the stirrups spacing reduced. However, this increase was not due to the increase in the CWP ratio but because of the decrease in spacing of stirrups.

**Figure 19:**
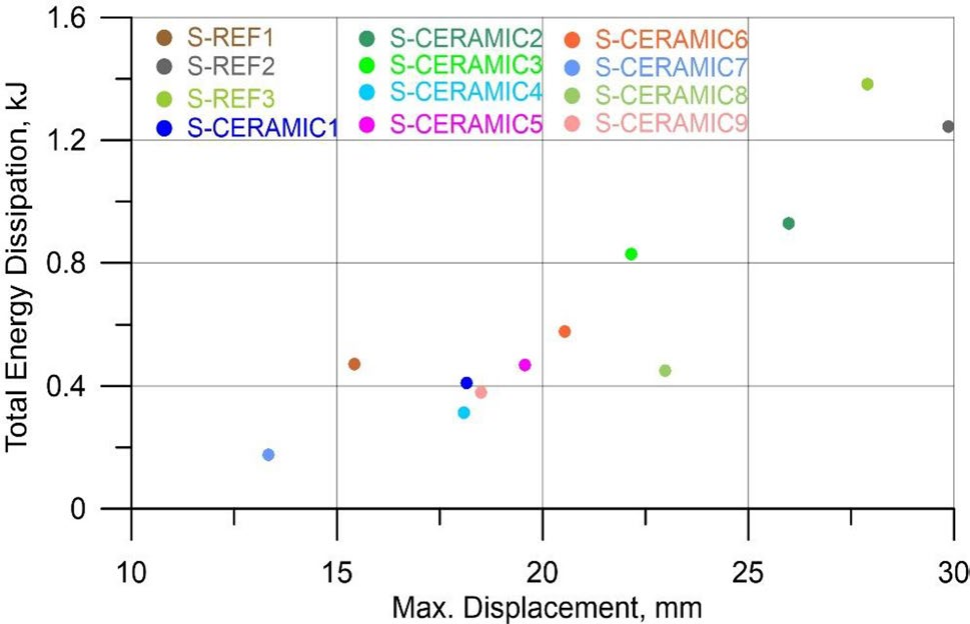
Assessment of energy dissipation for altered quantity of CWP.

Similar to the conclusions of the literature, it was identified that the factors affecting the energy dissipation were the cause of such ductility values^71^. To bear the increased loads operating on the construction, a structural element with a high degree of ductility is exposed to massive inelastic deformations. As an implication, the structural members undergo significant deformations before reaching the point of collapse^72^. The decrease in the resulting energy dissipation indicates that the structural element cannot withstand earthquakes largely because of a gradual failure mechanism. A comparison of the energy dissipation for altered quantity of CWP is depicted in Fig. 19.

## Calculation of shear capacity

Different empirical approaches for calculating the shear capacity of RCBs are available in the literature. In an empirical calculation close to the experimental results, parameters such as the cylinder concrete strength (*f*′_*c*_), RCBs' width (*b*_*w*_), shear span (*a*), stirrups spacing (*s*), reinforcement ratio (*ρ*), RCBs' effective depth (*d*), stirrups ratio (*A*_*v*_/*s*), and yield stress (*f*_*yd*_) of reinforcements should be known. The commonly used empirical formula in the shear capacity calculation, which is known to be close to the experimental results, is given in Eq. (1) ^73^.

1 $$V_{u} = 2.1746 \cdot \left( {f^{\prime}_{c} \cdot \rho \cdot \frac{d}{a}} \right)^{\frac{1}{3}} \cdot b_{w} d + \frac{{A_{v} f_{yd} }}{s}$$

**Figure 20:**
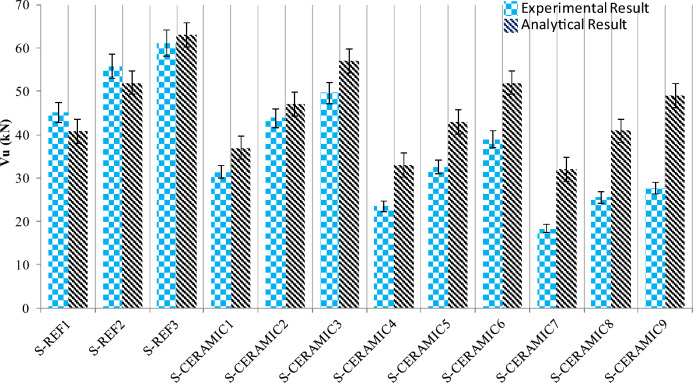
Experimental and analytical results for shear capacity.

It can be detected in Fig. 20 that as the CWP percentage in RCBs increased, the change in the shear capacity rose. As a result of the comparison, the analytical results were obtained with an approximation of up to 3.2% of the experimental results. The comparison of the experimental and analytical results is presented in Table 5. When Table 5 is examined, the analytical and experimental results diverged, as the CWP ratio in RCBs increased. This situation points out that the equation needs to be developed for the analytical calculations of CWP-added concretes.

**Table 5:** Comparison of experimental and analytical results for shear capacity.

Test samples	Experimental result (ER) for shear capacity (kN)	Analytical result for shear capacity (kN)	ER/AR
S-REF#1	45.11	40.7	1.10
S-REF#2	55.88	52	1.07
S-REF#3	61.03	63	0.97
S-CERAMIC#1	31.43	37	0.85
S-CERAMIC#2	43.87	47	0.93
S-CERAMIC#3	49.65	57	0.87
S-CERAMIC#4	23.51	33	0.71
S-CERAMIC#5	32.47	43	0.75
S-CERAMIC#6	38.94	52	0.75
S-CERAMIC#7	18.29	32	0.57
S-CERAMIC#8	25.51	40.9	0.62
S-CERAMIC#9	27.68	49	0.56

## Environmental values of CWP

More environmentally friendly and sustainable goods may be produced by recycling and using garbage that is solid in the production of building materials. These materials, however, must either be economically viable alternatives to currently used materials or have environmental advantages that justify their use. To assess the sustainability of CWP-mortar compared to regular mortar, it is necessary to choose metrics such as greenhouse gas emissions, production costs, and energy consumption related to mortar manufacture. The utilization of CWP-mortar is primarily influenced by these criteria, which are regarded as the main factors, despite the presence of other significant indications that also contribute. Samadi et al.^74^ evaluated the impact of substituting conventional Portland cement with a ceramic material on the emissions of greenhouse gases in a blended cement. The study found that higher levels of ground ceramic in the mortar samples led to a decrease in the emissions of greenhouse gasses. Along with a rise in ground ceramic from 20% to 40%–60%, the density of the gases emitted was also decreased from 92.9 kg/m^3^ to 77.5 kg/m^3^ and 46.8 kg/m^3^. During the production of one ton of blended cement, which consisted of 40% ground ceramic, the emission of 1 m^3^ of greenhouse gases occured, which represents a decrease of almost 37% compared to a conventional mixture^53^. Using CWP instead of typical aggregate will appear to have a huge environmental benefit, as can be demonstrated from the analyses above, even though the load-displacement curves are near to one other. Moreover, given the primary objective of employing CWP is to account for the environment, these statistics provide a significant perspective on quantifying the environmental advantages of adopting CWP.

## Conclusions

This study investigated the change in the shear capacity of RCBs produced with the CWP additives at different rates experimentally and analytically. Stirrups spacings in the specimens were chosen as 270 mm, 200 mm, and 160 mm. The findings obtained as results of the study can be summarized as follows:

- More than 10% CWP additive was determined to affect the concrete compressive strength negatively.
- The reference RCBs got the maximum load-carrying capaicty in all stirrups spacings (270 mm, 200 mm, and 160 mm). In the RCBs specimens, with stirrups spacings of 270 mm, 200 mm, and 160 mm, the CWP additives of 10%, 20%, and 30% improved the load-carrying capacity of RCBs, respectively, compared to the reference specimens. Consequently, adding CWP up to 10% to RCBs did not result in a significant reduction in the shear capacity. In other words, as CWP increased from 0% to 30%, the load-carrying capacity decreased between 30.3% and 59.4% compared to RCBs with stirrups spacing of 270 mm without CWP. However, reductions of 21.4%–54.3% and 18.6%–54.6% in the load-carrying capacity occurred, respectively, compared to RCBs with stirrups spacing of 200 mm and 160 mm without CWP.
- RCBs with different stirrups spacings, created with a 10% contribution of CWP, reduced the load-carrying capacity by 18.6% to 30.3%. This decrease reached 47.8% with a contribution of 20% CWP, and up to 54.3% with a contribution of 30% CWP. It was resulted that if the 10% CWP contribution was exceeded within the considered CWP ratios, the load-carrying capacity decreased too much. For different stirrups spacings, using 10% by weight of CWP may be recommended instead of cement.
- The maximum load value reached by the samples decreased as the CWP ratio increased in the samples with stirrups spacings of 160 mm, 200 mm, and 270 mm. However, with an increased CWP ratio in the samples with 160 mm stirrups spacing, RCBs reached the maximum load-carrying capacity at an earlier displacement value.
- When spacing between each stirrup was selected as 270 mm, it was observed that the maximum load-carrying capacity of RCBs reached at a similar displacement value as the CWP ratio increased.
- It was determined that the bending stiffness of RCBs reduced, as the quantity of CWP enhanced. In other words, the bending stiffness decrease was between 29.1% and 66.4% in the specimens with 270 mm stirrups spacing, between 36.3% and 20.2% in the specimens with 200 mm stirrups spacing, and between 10.3% and 36.9% in the specimens with 160 mm stirrups spacing.
- Evidence has shown that utilizing CWP can be regarded as an environmentally-friendly solution. This is because reusing CWP can substantially decrease CO_2_ emissions, conserve energy, lower overall electricity consumption, and reduce fuel usage. Consequently, this leads to the global availability of a sustainable and cost-effective construction material.

Consequently, in this study, the use of CWP, a mutual and inexpensive waste, in concrete was investigated with a series of experimental tests on RCBs. By the empirical research, the use of CWP up to 10% in RCBs is recognized as a cheap and ecologist approach and is suggested.

## Future work area

Additional testing and experimentation should be conducted on CWP to obtain its strength properties for use in typical or low-rise structural concrete applications. To get further insight into the workability, experimentation with different water/cement ratios may be done to determine the factors that affect the strength when sodium silicate is added. Moreover, the strength properties of CWP, which is also a pozzolanic material, may be further studied and researched. Studying CWP should be continued since it may help maintain the ecology and environment.

## Data Availability

The datasets used and/or analyzed during the current study are available from the corresponding authors on reasonable request.
